# A γ-Glutamyl Transpeptidase (GGT)-Triggered Charge Reversal Drug-Delivery System for Cervical Cancer Treatment: In Vitro and In Vivo Investigation

**DOI:** 10.3390/pharmaceutics15051335

**Published:** 2023-04-25

**Authors:** Jingxin Fu, Likang Lu, Manzhen Li, Yaoyao Guo, Meihua Han, Yifei Guo, Xiangtao Wang

**Affiliations:** 1 Institute of Medicinal Plant Development, Chinese Academy of Medical Sciences & Peking Union Medical College, Beijing 100193, China; 2 School of Traditional Chinese Materia Medica, Shenyang Pharmaceutical University, Shenyang 110000, China

**Keywords:** γ-glutamyl transpeptidase, charge reversal, paclitaxel, cervical cancer

## Abstract

Neutral/negatively charged nanoparticles are beneficial to reduce plasma protein adsorption and prolong their blood circulation time, while positively charged nanoparticles easily transverse the blood vessel endothelium into a tumor and easily penetrate the depth of the tumor via transcytosis. Γ-Glutamyl transpeptidase (GGT) is overexpressed on the external surface of endothelial cells of tumor blood vessels and metabolically active tumor cells. Nanocarriers modified by molecules containing γ-glutamyl moieties (such as glutathione, G-SH) can maintain a neutral/negative charge in the blood, as well as can be easily hydrolyzed by the GGT enzymes to expose the cationic surface at the tumor site, thus achieving good tumor accumulation via charge reversal. In this study, DSPE-PEG2000-GSH (DPG) was synthesized and used as a stabilizer to generate paclitaxel (PTX) nanosuspensions for the treatment of Hela cervical cancer (GGT-positive). The obtained drug-delivery system (PTX-DPG nanoparticles) was 164.6 ± 3.1 nm in diameter with a zeta potential of −9.85 ± 1.03 mV and a high drug-loaded content of 41.45 ± 0.7%. PTX-DPG NPs maintained their negative surface charge in a low concentration of GGT enzyme (0.05 U/mL), whereas they showed a significant charge-reversal property in the high-concentration solution of GGT enzyme (10 U/mL). After intravenous administration, PTX-DPG NPs mainly accumulated more in the tumor than in the liver, achieved good tumor-targetability, and significantly improved anti-tumor efficacy (68.48% vs. 24.07%, tumor inhibition rate, *p* < 0.05 in contrast to free PTX). This kind of GGT-triggered charge-reversal nanoparticle is promising to be a novel anti-tumor agent for the effective treatment of such GGT-positive cancers as cervical cancer.

## 1. Introduction

Statistics from the National Cancer Institute show that cervical cancer was persistently the second leading cause of cancer death in women aged 20–39 years in 2019, despite the popularization of HPV vaccination [1]. Compared to conventional therapies (surgery, chemotherapy, and/or radiotherapy), nanotechnology offers many possibilities in the delivery of chemotherapeutic drugs for cancer and has achieved considerable progress with the initial success of Doxil^®^ (PEGylated liposomal doxorubicin) [2].

Paclitaxel (PTX) is one of the most widely used broad-spectrum anti-cancer agents together with doxorubicin and cisplatin; PTX is considered one of the most successful natural anti-cancer drugs available and has been approved by the Food and Drug Administration for the treatment of breast, ovarian, and lung cancer, as well as Kaposi’s sarcoma [3,4]. As a highly lipophilic compound, the application of paclitaxel is restricted by its limited solubility, low bioavailability, and cosolvent-induced toxicity. Various nanocarrier systems, including micelles, liposomes, nanoparticles, bioconjugates, transfersomes, and polymeric dendrimers, have been employed to improve PTX’s solubility and reduce its undesired side effects [5,6]. 

Charge-reversal nanocarriers are neutral/negatively charged at various physiological conditions, such as in the blood circulation, to achieve long circulation time, but could be converted to positively charged by a certain stimulus, either exogenous (variations in temperature, ultrasound intensity, magnetic field, light, or electric pulses) or endogenous (changes in pH, redox gradients, or enzyme concentration) when they arrive at micro-vessels around the tumor [7,8,9]. Nanoparticles with cationic charge surfaces can much more easily transverse across the blood capillaries into the tumor [10] and also have stronger affinities to negatively charged tumor cell membranes; thus, charge-reversal can provide nanocarriers with both long blood circulation and deep tumor penetration [11], both of which are essential for tumor-targeted drug delivery. In contrast, although nanoparticles with an anionic charge surface can avoid protein adsorption, thus prolonging their blood circulation time, their weak interaction with negatively charged cell membranes leads to inefficient internalization by tumor cells [12]. Based on these, γ-Glutamyl transpeptidase (GGT)-triggered charge reversal drug-loading nanoparticles were constructed in this paper to achieve better anti-tumor therapeutic efficacy for PTX.

**Figure 1:**
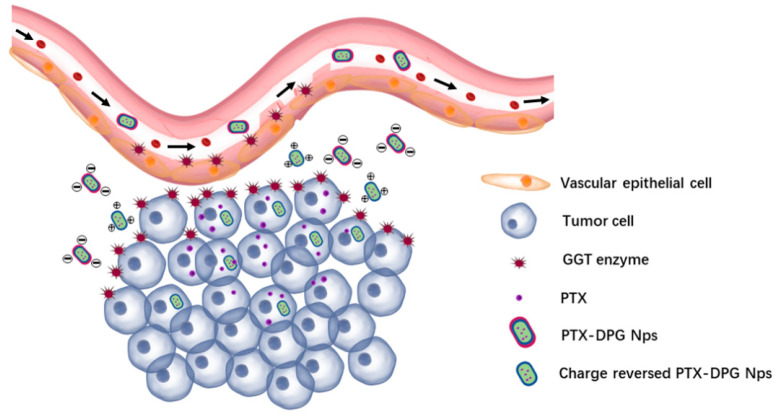
The schematic diagram for the charge reversal of PTX-DPG NPs at the tumor site and their infiltration from microvessels into the tumor.

GGT is usually regarded as a nonspecific marker of liver dysfunction and has a vital role in the extracellular catabolism of GSH (the major antioxidant in mammalian cells). The serum levels of GGT may be associated with a wide range of disease outcomes, including cancer [13]. Increased expression of GGT has been found in several human tumors (including HEK293 [14], Hela [15], HepG2 [16], MCF-7 [17], and U87MG cell lines [14]) for cleaving extracellular glutathione and providing cells with amino acids necessary for intracellular biosynthesis to meet the requirements of the vigorous metabolism of tumor cells [18]. GGT is overexpressed on the external surface of the endothelial cells and metabolically active tumor cells at the periphery of the blood vessels (Figure 1), and the activity of GGT on the tumor cellular membrane surface is ten-fold higher than that of GGT in the blood [19]. It has been proven that the overexpressed GGT on the vascular endothelial cell or tumor cell triggers the γ-glutamyl transfer reactions of glutathione to achieve deep penetration of nanocarriers modified with glutathione [20] or containing γ-glutamyl moieties [17]. The near-infrared (NIR) fluorescent probe with the GGT-recognizable substrate and c-RGD can effectively differentiate the tumor cells from GGT-positive and αvβ3-deficient normal cells [14].

In this work, DSPE-mPEG2000-NH2 was conjugated with the −COOH group of the protected GSH tripeptide by amide condensation, followed by the removal of the protective groups. The DSPE-PEG2000-GSH (DPG) is nearly neutral, but its terminal GSH peptide contains γ-glutamyl amide that is sensitive to and can be hydrolyzed by GGT enzyme, which is highly expressed in the tumor, leading to the exposure of the amino group and positive charge of the whole system.

DPG and PTX were made into nanoparticles via the anti-solvent precipitation method. The resultant rod-like PTX-DPG nanoparticles are characteristic of negative charge. However, in the presence of the GGT enzyme, the zeta potential of PTX-DPG NPs changed from −5.24 mV to neutral within 1 h and converted to be cationic in the subsequent hours, demonstrating the charge reversal property of PTX-DPG NPs. It is reported that cationization could effectively induce adsorption-mediated transcytosis of nanoparticles and realize the deep penetration into multilayered cell culture. This property enables the PTX-DPG NPs to be widely and deeply distributed in tumors and leads to remarkably improved anti-tumor efficacy in vivo.

## 2. Materials and Methods

### 2.1. Materials

DSPE-mPEG_2000_-NH_2_ was provided by Shanghai ToYong Bio. Tech. Inc. (Shanghai, China). Boc-γ-Glu (OtBu)-Cys (Trt)-Gly [Boc-γ-GSH] was from Nanjing TG peptide Biotechnology Co., LTD (Nanjing, China). Paclitaxel (PTX) was bought from Jiangsu Aikang Biomedical Research and Development Co. Ltd. (Nanjing, China). Trifluoroacetic acid (TFA) was purchased from Shanghai Macklin Biochemical Co., Ltd. (Shanghai, China). Dichloromethane (super dry solvent, with molecular sieves, water ≤ 30 ppm) was bought from J&K Scientific, Inc. (Beijing, China). Triisopropylsilane (97.5%) (Tis) was provided by J&K Scientific, Inc. (Beijing, China). 1-Ethyl-3-(3-dimethylaminopropyl) carbodiimide hydrochloride (EDCI) was from Meryer (Shanghai) Chemical Technology Co., Ltd. (Shanghai, China). N-Hydroxy succinimide (NHS) was purchased from Shanghai Yuanye Bio-Technology Co., Ltd. (Shanghai, China). DiR iodide [1-1-dioctadecyl-3,3,3,3-tetramethlindotricarboc-yanine iodide] (DiR) was purchased from AAT BioQuest (Sunnyvale, CA, USA). Acetonitrile (HPLC grade) was from Fisher Scientific (Pittsburgh, PA, USA). Paclitaxel injection (PTX injection) was obtained from Beijing Union Pharmaceutical Factory (Beijing, China). Deionized water was used during the experiments.

### 2.2. Animals and Cell Line

The Hela (cervical carcinoma) cell line was purchased from China Infrastructure of Cell Line Resource. The cells were cultured in DMEM medium containing 10% fetal bovine serum (FBS, Gibco, Waltham, MA, USA); 100 U/Ml streptomycin and penicillin (Gibco, Waltham, MA, USA) were added for the prevention of bacterial contamination. The cells were kept in a 5% CO_2_ atmosphere at 37 °C constantly.

Female nu/nu BALB/c healthy mice weighing 20 ± 2 g (6–8 weeks old) (SPF grade) were purchased from SPF (Beijing, China) Biotechnology Co., Ltd. The experimental animals were adapted to the environment of SPF-class housing of laboratory at least 7 days before experimentation and were provided ad libitum feeding. 

All animal experiments were conducted according to the guidelines for Ethical and Regulatory Animal Experiments Stipulated by the Institute of Medicinal Plant Development (IMPLAD).

### 2.3. Synthesis of DSPE-mPEG_2000_-GSH(DPG)

**Figure 2:**
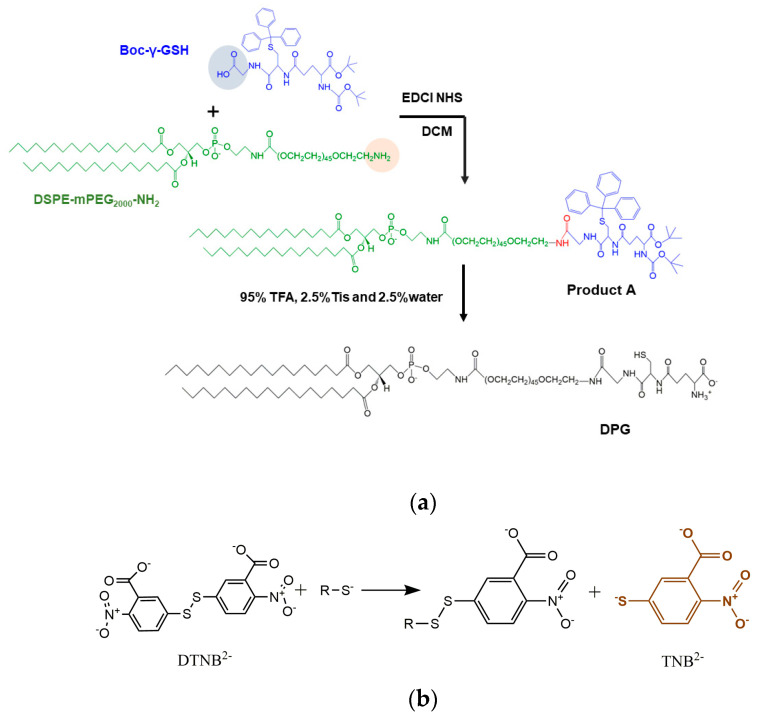
The synthetic route of DPG (**a**) and the reduction of Ellman’s reagent (**b**).

The synthesis of DPG was carried out as shown in Figure 2a. Firstly, Boc-γ-GSH (26.4 mg, 0.037 mmol), EDCI (14.13 mg, 0.074 mmol), and NHS (8.51 mg, 0.074 mmol) were co-dissolved in 5 mL anhydrous dichloromethane and stirred for 4 h at 0 °C in an ice bath. After activation, DSPE-mPEG_2000_-NH_2_ (104 mg, 0.037 mmol) and one drop of triethylamine were added and reacted at room temperature for 24 h. When the reaction was completed, DCM was removed by rotary evaporation at 45 °C, and the mixture was washed three times with saturated NaCl solution to generate Product A (Figure 2a). Then 95% TFA, 2.5% Tis, and 2.5% water were added to deprotect the Boc and Trt groups from Product A. After stirring overnight, the solution was concentrated by rotary evaporation, and then the final product was precipitated by a large amount of ice ether, followed by centrifugation at 8000 rpm for 10 min to collect DPG (yield ~72.58%). The final product was dried under freeze-drying overnight and characterized by ^1^H NMR. ^1^H NMR (600 MHz, Chloroform-d): δ0.88 (t, 6 H), 1.26 (s, 56 H), 1.66 (s, 4 H), 2.32 (m, 4 H), 3.16 (s, 1 H), 3.37 (s, 1 H), 3.5–3.95 (m, 184 H), 4.0–4.5 (m, 8 H), 5.26 (s, 1 H), and 6.76 (s, 1 H).

### 2.4. GSH Grafting Ratio Determination

Ellman introduced 5,5’-dithio-bis-(2-nitrobenzoic acid), also known as DTNB, as a versatile water-soluble compound for quantitating free sulfhydryl groups in a solution [21]. DTNB solution presents a yellow-colored product that could be used in quantitative analysis when it reacts with sulfhydryl (Figure 2b). Sulfhydryl groups may be estimated by comparison to a standard curve of L-cysteine, which is composed of known concentrations of a sulfhydryl-containing compound. A series of concentrations of L-cysteine was mixed with Ellman’s reagent, incubated at room temperature for 15 min, and then the absorbance at 412 nm was measured using a UV spectrophotometer to plot a standard curve. Each molecule of DPG contains one molecule of −SH, which is the same as L-cysteine. 52.4 mg DPG was dissolved in 5.24 mL methanol and mixed with 50 μL Ellman’s reagent, incubated for 15 min, and then the absorbance at 412 nm was determined and the -SH concentration was obtained from the standard curve to compare with the DPG concentration to calculate the GSH grafting ratio.

### 2.5. Critical Micelle Concentration (CMC) Determination of DPG

Pyrene is a commonly used hydrophobic fluorescent probe with a fluorescence spectrum of five peaks, among which the ratio of fluorescence intensity at 373 nm (I1) against that at 384 nm (I3) (I1/I3) is very sensitive to the polarity of the microenvironment. After the formation of micelles, the pyrene probe enters the hydrophobic microregion of micelles from the aqueous phase, and the change in the microenvironment polarity decreases the value of I1/I3 significantly. The corresponding surfactant concentration at this abrupt change is determined as a CMC value [22].

Different concentrations (20, 18, 15, 12, 10, 9, 8, 7, 6, 5, 4, 3, 2, 1.5, 1, 0.8, 0.5, 0.2, 0.1, 0.08, 0.05, 0.01, 0.005, and 0.001 μg/mL) of DPG micelles were mixed with pyrene acetone solution (0.1 mM) for 2 h to obtain the fluorescence spectra of the mixture with different concentrations (Ex wavelength = 335 nm). The CMC value of DPG was obtained by calculating the fluorescence intensity ratio (I1/I3).

### 2.6. Preparation of PTX-DPG NPs

**Figure 3:**
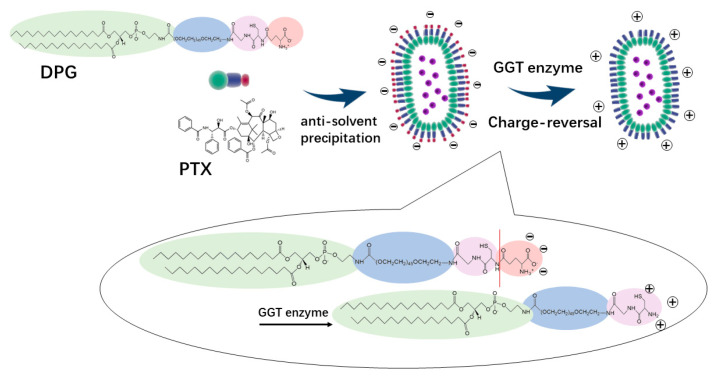
The schematic diagram for the formation of PTX−DPG−NPs and their charge−reversal triggered by GGT enzyme.

The encapsulation of PTX by DSPE-PEG_2000_-GSH (DPG) was carried out through the method of anti-solvent precipitation (Figure 3). PTX and DPG with a mass ratio of 1:1 were co-dissolved in methanol, and then the mixture was injected into an appropriate volume of deionized water under constant ultrasonic conditions, drop by drop (250 W, 25 °C). The organic solvent was removed under vacuum by rotary evaporation at 45 °C. Then, the resultant nanosuspensions were homogenized (~1560 bar) at room temperature 10 times to obtain the final PTX-DPG NPs.

When DiR or coumarin 6 (Co-6) was co-dissolved in methanol together with PTX and DPG (the mass ratio of DiR or Co-6 with PTX being 1:40), the same method as above produced DiR-labeled PTX-DPG NPs or Co-6 labeled PTX-DPG NPs. When DiR was co-dissolved in methanol with DPG (the mass ratio of DiR to DPG as 1:40), in the absence of PTX, the same method as above produced DiR-labeled DPG micelles.

### 2.7. Morphology of PTX-DPG NPs

The morphology of DPG-PTX NPs was observed by Scanning Electron Microscopy (SEM; S-4800, Hitachi Limited, Tokyo, Japan) and Transmission Electron Microscopy (TEM; JEM-1400, JEOL, Tokyo, Japan). Briefly, diluted DPG-PTX NPs were placed on the matrix and air-dried. After sputter-coating with Au/Pd for 1 min, the samples were observed by SEM at an accelerating potential of 30 mV. Another drop of diluted DPG-PTX NPs was added to the copper grid (300 mesh), air-dried, and then dyed with uranyl acetate for an appropriate time. Then, the morphology was observed under TEM at an accelerating voltage of 120 kV.

### 2.8. HPLC Determination

The concentration of PTX in nanoparticles was measured by the HPLC system (DIONEX Ultimate 3000, Waltham, MA, USA). A Symmetry C18 column (250 mm × 4.6 mm, 5 μm, Venusil) was used at 25 °C for chromatographic separation. The mobile phase constituted 75% acetonitrile and 25% water (3:1, *v/v*) at a flow rate of 0.8 mL/min. The detection wavelength UV was 240 nm.

### 2.9. Drug Loading Content

Lyophilized PTX-DPG NPs were accurately weighed, dissolved in acetonitrile, centrifuged at 5000 rpm for 5 min, and the supernatant was injected in HPLC for the determination of PTX concentration. The drug loading content (*DLC*) of PTX-DPG NPs was calculated as follows:

$$DLC\left(\%\right)=\frac{V\cdot C}{W}\times 100\%$$

(*V*: Volume of an acetonitrile solution of lyophilized PTX-DPG NPs powder; *C*: Concentration of PTX; *W*: Weight of the lyophilized powder of PTX-DPG NPs).

### 2.10. Charge-Reversal Examination

As the tumor site highly expresses GGT enzyme up to 10 U/mL [19], 1.8 mg of GGT (27.2 U/mg) was weighed and dissolved in 5 mL of pre-warmed 37 °C PBS aqueous solution (pH = 7.4) to obtain 10 U/mL of GGT solution (pH = 7.4). 10 U/mL of GGT solution was diluted into 0.05 U/mL to simulate the activity of the GGT enzyme in the blood. 1 mL PTX-DPG nanosuspensions and 1 mL GGT solution of different concentrations were mixed and incubated at 37 °C. Zeta potential changes were determined by DLS at specific time intervals.

### 2.11. Surface Element Analysis

The surface chemical composition of PTX powder and PTX-DPG NPs were investigated by Scanning electron microscopy (SEM) with Energy Dispersive Spectrometer (SEM-EDS; S-4800, Hitachi Limited, Tokyo, Japan). These samples were sputter-coated with a conductive layer of gold-palladium (Au/Pd) for 1 min. An accelerating potential of 30 mV was used for the observation and analysis.

### 2.12. Stability of PTX-DPG NPs On-Shelf or in Physiological Media

PTX-DPG NPs were stored at 4 °C for 25 days. Their particle size and PDI value were measured at 0, 1, 3, 5, 7, 14, and 25 days. Each sample was performed in triplicate. PTX-DPG NPs were respectively incubated with the same volume of 1.8% NaCl, 10% glucose, 2 × PBS (pH 7.4) (1:1, *v/v*), and plasma or four times the volume of simulated gastric fluid (SGF, 1% pepsin in 1 mol/L diluted HCl) and simulated intestinal fluid (SIF, 1% pancreatin in pH 6.8 PBS, 0.01 M) (1:4, *v/v*) at 37 °C. The size and particle size distribution were determined by DLS at specific time intervals. Each sample was carried out in triplicate.

### 2.13. Hemolytic Test

Red blood cells (RBCs) were obtained from healthy mice; whole blood was centrifuged, washed several times with normal saline, and diluted to 4% (*v/v*) RBCs suspensions with normal saline. The 4% RBCs suspensions were mixed with deionized water (as the positive control group), normal saline (as the negative control group), and different concentrations (1.8, 1, 0.5, 0.25, 0.1 mg/mL) of PTX-DPG NPs (as the test groups) at the volume ratio of 1:1. After incubation at 37 °C for 4 h, the mixture was centrifuged at 5000 rpm for 3 min to observe its hemolytic effect.

### 2.14. Differential Scanning Calorimetry (DSC) Characterization

DSC thermal profile was obtained using a differential scanning calorimeter (DSC Q200 V24.4 Build 116). A suitable amount of PTX powder, DPG powder, lyophilized PTX-DPG NPs powder, and the physical mixture (DPG:PTX = 1:1 *w/w*, according to the formulation of PTX-DPG NPs) were sealed in a standard aluminum pan and detected from 0 to 350 °C (10 °C/min, a nitrogen atmosphere).

### 2.15. X-ray Diffraction (XRD) Measurements

X-ray diffraction (XRD) measurements of PTX powder, DPG powder, lyophilized PTX-DPG NPs powder, and the physical mixture (DPG:PTX = 1:1, *w/w*, according to the formulation of PTX-DPG NPs) were performed using X-ray diffractometer (DX-2700, China) with Cu-Kα radiation generated at 100 mA and 40 kV. Samples were scanned over an angular range of 3–90° of 2θ°, with a step size of 0.02° and a count time of 3 s per step. Samples were kept rotating at 30 rpm during the analysis.

### 2.16. Drug Release Profile

The in vitro drug release of PTX from PTX-DPG NPs was conducted using the dialysis method (molecular weight cut-off 8 k–14 kDa) at 37 °C under continuous stirring (200 rpm). 1 mL PTX-DPG NPs was sealed in a dialysis tube and then immersed in 50 mL PBS (pH 5.7, 6.4, and 7.4, containing 2% Cremophor EL) mimicking different physiological environments. At the predetermined time intervals, 1 mL of the dialysate was collected for HPLC analysis and replenished with an equivalent amount of fresh-release medium. The release medium was renewed every 24 h. The above experiment was performed in triplicate.

### 2.17. MTT Assays

Multiple tumor cell lines, including Hela cells, overexpress the γ-GGT enzyme [14,15,16,23,24]. MTT assay was carried out to examine the in vitro cytotoxicity of PTX-DPG NPs against the Hela cell line using free PTX as a control. Hela cells (5000 cells/well) were seeded in 96-well plates and incubated at 37 °C and in a 5% CO_2_ atmosphere for 24 h. Different concentrations of PTX-DPG NPs (diluted in DMEM medium) were added (150 μL per well) and incubated for 72 h. Then 20 μL of MTT solution (5 mg/mL) was added to each well and incubated for another 4 h. Then, the medium was removed and 150 μL of DMSO was added to dissolve the formazan crystals. The absorbance value of the supernatant in each well was measured at 570 nm using the ELISA plate reader. The *cell viability rate* was calculated according to the following formula:

$$Cell\;viability\;rate\left(\%\right)=\left(1-\frac{{OD}_{t}}{{OD}_{n}}\right)*100\%$$

(where *OD_t_* means the absorbance value of the test groups, *OD_n_* means the absorbance value of the negative control groups).

### 2.18. Cellular Uptake

Hela cells (5 × 10^5^ cells per well) were inoculated in a 24-well plate and cultured in a DMEM medium for 24 h at 37 °C in the presence of 5% CO_2_, and then, PTX-DPG NPs (PTX equivalent concentration 10 μg mL^−1^) were added into the 24-well plate. They were washed, dyed with DAPI, and fixed with 0.4% paraformaldehyde after 0.2, 1, 2, and 6 h, respectively. The results were recorded using an inverted fluorescence microscope.

### 2.19. In Vivo Anti-Tumor Efficacy and Safety Evaluation

Female nu/nu BALB/c mice weighing 20 ± 2 g bearing Hela tumors were chosen for the in vivo anti-tumor efficacy investigation. 0.2 mL Hela cell suspension (1 × 10^6^ cells) was injected into the right armpit subcutaneously of mice to construct Hela-tumor bearing mice model. When the tumor volume reached 100 mm^3^, the Hela tumor-bearing mice were randomly divided into 4 groups (6 mice in each group). The negative control group was intravenously injected with 0.2 mL of normal saline, the positive control group was administrated with 0.2 mL of PTX injection (8 mg/kg). PTX injection (8 mg/kg) was diluted from the marked Paclitaxel injection purchased from the Beijing Union pharmaceutical factory by normal saline. The other test groups were intravenously injected with 8 and 16 mg/kg of PTX-DPG NPs, respectively (the equivalent PTX dose). All the above groups were intravenously administrated through the tail vein every 2 days for 5 times. Tumor volume and body weight of mice in each group were measured during the whole experimental process. The tumor volume was calculated by the formula V = (a × b^2^)/2.

At the end of the experiment, the blood was collected for each mouse, then all the mice were sacrificed, and the tumors, livers, and spleens were dissected and weighed to calculate the tumor inhibition rate (*TIR*%), liver index rate (*LIR*%), and spleen index rate (*SIR*%) according to the following formulas:

$$TIR\left(\%\right)=\left(1-\frac{W_{t}}{W_{n}}\right)\times 100\%$$

(*W_t_* is the mean tumor weight of mice in test groups and *W_n_* is the mean tumor weight of mice in the negative control group)

$$LIR=\sum_{i=1}^{9}\begin{array}{c} \frac{W_{l}}{W_{b}}÷9 \end{array}$$

(*W_l_* is the liver weight and *W_b_* is the body weight)

$$SIR=\sum_{i=1}^{9}\begin{array}{c} \frac{W_{s}}{W_{b}}÷9 \end{array}$$

(*W_s_* is the spleen weight and *W_b_* is the body weight).

All the tumor tissues were stained with hematoxylin and eosin (H&E) to evaluate the effect of the therapeutic efficacy. The IHC staining of Ki67 was tested to investigate the percentage of proliferous tumor cells positively stained in the examined field. The blood serum was separated to detect the expression levels of tumor cytokines (TNF-α and IFN-γ) by ELISA Kits (Fankewei, Shanghai, China) according to the manufacturer’s protocol.

### 2.20. Biodistribution Evaluation

To observe the dynamic biodistribution of DiR-labelled DPG micelles and PTX-DPG NPs, 6 Hela tumor-bearing mice (tumor volume of ~1000 cm^3^) were injected through the tail vein (3 mice in each group). They were whole-body imaged at 0.2, 0.5, 1, 2, 4, 6, 8, 12, and 24 h post-dose using the IVIS Living Image software (version 4.4, Caliper Life Sciences, Hopkinton, MA, USA). Then the mice were sacrificed, and the tumors and the major organs such as hearts, livers, spleens, lungs, and kidneys, were also imaged to measure the fluorescence intensity in these tissues.

### 2.21. Statistical Analysis

Statistical analysis of the experimental data was performed using Statistical Package for the Social Sciences software, and IC_50_ values were calculated by GraphPad Prism software, version 6.01 (GraphPad Software, La Jolla, CA, USA). In vitro and in vivo results were analyzed by *t*-test and one-way analysis of variance. The value of *p* < 0.05 was considered statistically significant.

## 3. Results and Discussion

### 3.1. Synthesis and Characterization of DPG

**Figure 4:**
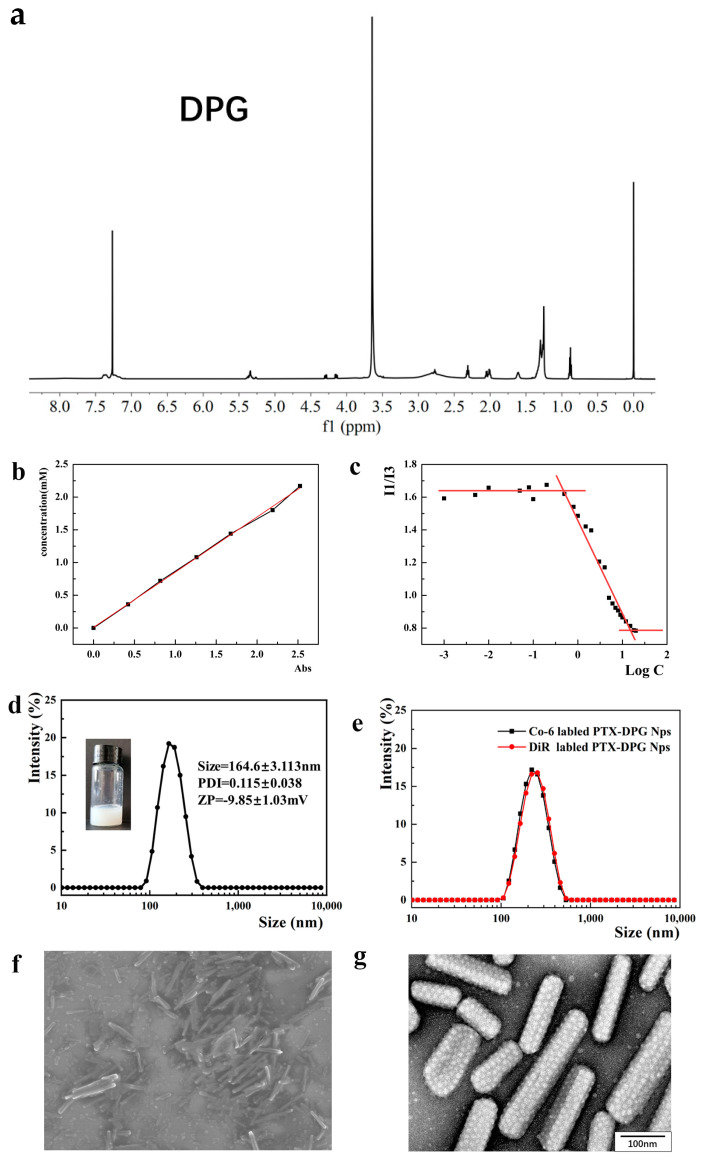
(**a**) 1H NMR of DPG. (**b**) Standard curve of L−cysteine. (**c**) Critical micelle concentrations (CMC) of PTX−DPG NPs measured by fluorescence spectrophotometer using pyrene as a probe. (**d**) Particle size distribution of PTX−DPG NPs. (**e**) Particle size distribution of Co−6 and DiR−labelled PTX−DPG NPs. (**f**) Scanning Electron Microscopy image of PTX−DPG NPs. (**g**) Transmission Electron Microscopy image of PTX−DPG NPs (Scale: 100 nm).

Amphiphilic molecules modified by Boc-γ-GSH were synthesized via the amide condensation from DSPE-mPEG_2000_-NH_2_ and Boc-γ-GSH (Figure 2), and the Trt, Boc, and tert-butyl ester protective groups on the resultant DPG were removed by the TFA treatment. The final product was purified by the diethyl ether precipitation method (73.98% in the yield). The characteristic peaks of DSPE-mPEG_2000_-NH_2_ were shown in the ^1^H NMR spectrum of DPG, the peaks of protective groups vanished after dealing with TFA. The characteristic peak of DSPE-mPEG2000 at 0.88 ppm still existed in the diagram of ^1^HNMR of DPG (Figure 4a).

Quantitative determination of DPG was carried out by Ellman’s reagent [25], a traditional method for the quantification of thiols or cysteine residues of proteins using DTNB by George L. Ellman. The standard curve of L-cysteine/methanol solution was y = 0.8435x + 0.0315 (y means the concentration of -SH and x means the absorbance at 412 nm, with the linearity range of 0.36~2.17 mM, R2 = 0.999) (Figure 4b), on basis of which the concentration of DPG/methanol solution could be determined as there was only one single cysteine residue in DPG molecule. The absorbance of the DPG solution at 412 nm was 1.731, which meant the −SH concentration of the DPG solution was 2.94 mM according to the standard curve. Since the DPG was 3.30 mM in the solution, the GSH grafting ratio in DPG was calculated to be 89.09%.

### 3.2. Critical Micelle Concentration (CMC) Determination of DPG

It was reported that the CMC of DSPE-mPEG was below 1 × 10^−5^ mol/L, namely below 0.028 μg/mL [26]. However, the CMC of DPG was determined to be about 6.35 μg/mL (=0.0016 mM) by the pyrene probe method (Figure 4c), indicating that DPG could still easily form micelles and load hydrophobic drugs; More than 220 times higher CMC than DSPE-mPEG meant that the GSH moiety of DSPE-PEG-GSH (DPG) was hydrophilic and thus tended to be exposed at the surface of DPG micelles or nanoparticles, benefiting the charge reversal in vitro and in vivo.

### 3.3. Preparation and Characterization of PTX-DPG NPs

PTX-DPG NPs were prepared by an anti-solvent precipitation method and displayed a mean particle size of 164.6 ± 3.113 nm with a narrow distribution (PDI = 0.115 ± 0.038) and a negative zeta potential of −9.85 ± 1.03 mV (Figure 4d). Co-6-labeled PTX-DPG NPs and DiR-labeled PTX-DPG NPs displayed slightly increased average particle sizes of 220 nm and 255 nm, respectively (Figure 4e). The drug-loaded content of PTX in DPG-PTX NPs was 41.45 ± 0.7% measured by HPLC.

The morphology of PTX-DPG NPs was observed via SEM and TEM, both showing that PTX-DPG NPs were uniformly dispersed as regular nanorods (Figure 4f,g), with the length being about 200 nm and the width being 50–100 nm (Figure S1). However, the particle size of the PTX-DPG NPs measured by SEM and TEM was smaller than that measured by DLS because the electron microscope displayed dry nanoparticles whereas DLS measured the hydrated nanoparticles with the hydrophilic PEG shell fully extended in an aqueous solution.

As for the formation of PTX-DPG nanorods or rod-shaped nanoparticles, we guess it is mainly because PTX itself is a needle-like or rod-like crystal habit. It could be seen from the published papers that for PTX nanoparticles, low drug-loading content of PTX tended to result in a spherical shape [27,28], while a high drug-loading content of PTX tended to lead to a nanorod shape [29,30]. This is mainly because PTX has a strong self-crystallization tendency.

During the preparation of PTX-DPG-NPs using anti-solvent precipitation, after the methanol solution of PTX and DPG was dropped in water, and the solvent methanol was quickly diffused into the bulk water, then PTX and DPG began to self-assemble driven by the hydrophobic interaction into a transitional microphase with a preliminary core-shell structure, for which the PEG chain and the terminal GSH peptide formed the shell while PTX and the DSPE moiety formed the core. In the core of this transitional core-shell microphase, there is a large amount of free PTX and a small amount of DSPE segment. Along with the further diffusion of methanol, PTX molecules crystallized in the inner core and tended to form rod-like crystals, as PTX had such a crystal habit, and the presence of a small amount of DSPE segment failed to affect the crystallizing process of PTX.

### 3.4. Charge-Reversal Examination

**Figure 5:**
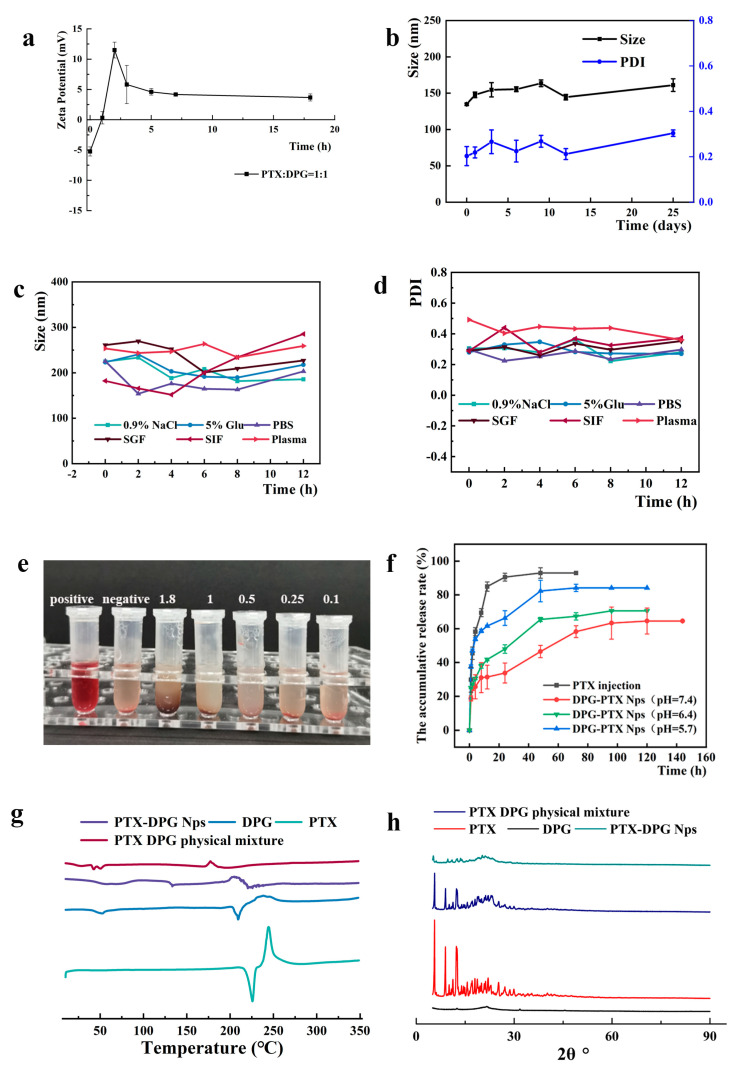
(**a**) Charge-reversal property of PTX−DPG NPs in 10 U/mL γ−GGT solution. (**b**) Storage stability of PTX−DPG NPs at 4 °C for 25 days. (**c**) The change in particle size of PTX−DPG NPs in various physical medium. (**d**) The change in the size distribution of PTX−DPG NPs in various physical medium. (**e**) In vitro analysis of hemolytic properties. (From left to right, the positive group, the negative group, 1.8 mg/mL, 1 mg/mL, 0.5 mg/mL, 0.25 mg/mL, 0.1 mg/mL PTX−DPG NPs). (**f**) Drug release profile of PTX injection (at pH 7.4) and DPG−PTX NPs (at different pH values). (**g**) DSC pattern of PTX−DPG NPs. (**h**) XRD pattern of PTX−DPG NPs.

When incubated with 10 U/mL GGT solution, the zeta potential of PTX-DPG NPs was changed from −5.24 mV to neutral within 1 h and converted to cationic in the subsequent hours (Figure 5a). However, when incubated with 0.05 U/mL GGT solution or PBS (pH = 7.4 ± 0.2) at 37 °C for 24 h, their zeta potential changed to −0.352 ± 0.584 mV and −0.395 ± 0.303 mV, indicating the maintenance of a neutral surface in the low GGT environment. This meant when circulating in the blood, where there is a low concentration of GGT enzyme (0.05 U/mL), PTX-DPG NPs are slightly negatively charged or neutral, which benefits long circulation. However, in the microvessels around the tumor or in the tumor microenvironment, where there is a high level of GGT enzyme, PTX-DPG NPs will be converted to cationic, thus beneficial to tumor penetration and anti-tumor efficacy.

### 3.5. Surface Element Analysis

The surface element analysis of PTX-DPG nanorods (Table 1) demonstrated the presence of a higher amount of S element (1.17% vs. 0%) and O element (49.37% vs. 22.09%) in contrast to that of PTX bulk powder, suggesting the PEG chains and GSH peptide were mostly distributed on the surface of PTX-DPG nanorods, due to the outside PEG chain showing higher oxygen content and the GSH peptide showing higher sulfur content.

### 3.6. Stability of PTX-DPG NPs on the Shelf or in Physiological Media and Hemolysis Test

During the 25 days of storage at 4 °C, the average particle size of PTX-DPG NPs maintained at about 160 nm and the PDI value was basically unchanged, though there was a slight fluctuation (Figure 5b). This showed that PTX-DPG NPs had good stability on the shelf.

Drug-loading nanoparticles need to maintain their nanoscale particle size in the physiological environment for in vivo drug delivery, as some nanoparticles are stable in deionized water but show significant particle size enlargement or even form aggregates or precipitates, thus failing to deliver the drug in vivo. As seen in Figure 5c,d, PTX-DPG NPs showed good stability in normal saline, PBS (pH = 7.4), 5% glucose, and plasma during the whole process of incubation, with quite a similar particle size and PDI value, and no turbidity or aggregation was observed at all. While in the SGF and SIF, there was a little higher fluctuation (50 nm and 100 nm, respectively, at most) during the 12 h of incubation. All in all, PTX-DPG NPs were stable in various physiological media. Since PTX-DPG NPs displayed the smallest average particle size and PDI value in PBS, PTX-DPG NPs in PBS were used in the subsequent experiment and in vivo study. In the hemolysis test, no rupture of RBC was observed after incubation of mouse red blood cells (RBC) with PTX-DPG NPs at different concentrations of equivalent PTX (Figure 5e), indicating good safety of PTX-DPG NPs for intravenous administration.

### 3.7. Differential Scanning Calorimetry and X-ray Diffraction

The DSC pattern (Figure 5g) shows that there were melting absorption and decomposition exothermic peaks of paclitaxel appearing at 225.78 and 244.85 °C, respectively, indicating PTX is crystalline. However, neither PTX-DPG NPs nor the physical mixture displayed similar melting absorption or decomposition exothermic peaks as PTX did, indicating it was difficult for DSC to recognize the presence of the crystalline form of PTX in PTX-DPG NPs. This was probably because DPG was a tri-block polymer and all three blocks had different melting behaviors. Especially the PEG2000 block showed a melting point of about 52 °C, thus it may at least partially dissolve PTX in the physical mixture during the heating process of the DSC determination.

The XRD patterns (Figure 5h) of the PTX powder, DPG powder, and physical mixture of PTX\DPG and PTX-DPG NPs were examined under the same conditions. There were similar sharp diffractions in the curves of PTX powder, physical mixture, and PTX-DPG NPs. Although the presence of DPG and the preparation process of PTX-DPG NPs weakened the signal of the diffraction peaks of the crystallinity of the co-existing PTX, the crystalline form was still maintained in PTX-DPG NPs.

The feeding ratio of PTX and DPG was 1:1 (*w/w*) for the preparation of PTX-DPG NPs, during which PTX has a big possibility to form crystals as PTX had a strong self-crystallization. In this study, PTX-DPG NPs presented a rod-like morphology as observed by TEM and SEM, and in most reports, PTX was in crystalline form in PTX nanorods [30,31,32]. The above analysis demonstrated that PTX should be in crystalline form in PTX-DPG NPs.

### 3.8. Drug Release Profile

The drug release experiment was performed in PBS at different pH values (5.7, 6.4, and 7.4), respectively simulating the normal tissues, tumor microenvironment, and endo/lysosomal microenvironment. As illustrated in Figure 5f, PTX was quickly released from the PTX injection and reached 84.93% within 12 h. This was because PTX injection was PTX solution in the mixture of ethanol and cremophor EL, and thus PTX could quickly diffuse across the dialysis tubing into the release medium. Differently, DPG-PTX NPs demonstrated a much slower in vitro drug release characteristic of pH dependence, with a slow release at pH 7.4, a quicker release at pH 6.4, and an even quicker release at pH 5.7 (Figure 5f). The corresponding cumulative release rates within 48 h reached 46.47%, 65.45%, and 82.30%, respectively. This pH-responsive drug release property would help DPG-PTX NPs reduce drug leakage in circulation while timely releasing the encapsulated PTX in the tumor to exert anti-tumor efficacy. The seeming burst release was because of the PBS used in this experiment, which contained 2% Cremophor EL to boost the drug release from DPG-PTX NPs. In PBS alone, no PTX release could be detected at all, even during the 72 h dialysis.

### 3.9. In Vitro Anti-Tumor Efficacy and Cell Uptake

**Figure 6:**
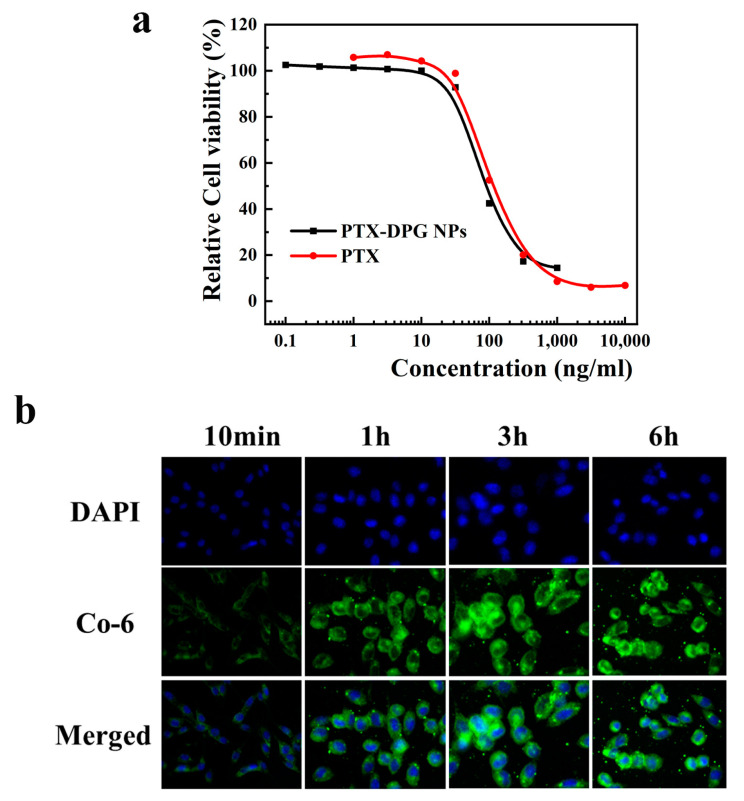
(**a**) MTT assay of PTX-DPG NPs and free PTX in the Hela cell line. (**b**) Cellular uptake of Co-6-labeled PTX-DPG NPs in the Hela cell line at a different time point (Blue-DAPI, Green- Co-6-labelled PTX-DPG NPs).

MTT assay was applied to evaluate the in vitro anti-tumor activity of PTX-DPG NPs. As illustrated in Figure 6a, free PTX and PTX-DPG NPs showed a dose-dependent inhibitory effect on the growth of the Hela cells, with the IC_50_ being 95.17 ng/mL and 73.88 ng/mL, respectively. PTX-DPG NPs showed a little higher cytotoxicity against the Hela cells than free PTX.

The cellular uptake of PTX-DPG NPs was also performed using coumarin-6 (Co-6)-labeled PTX-DPG NPs. As shown in Figure 6b, the cellular uptake of PTX-DPG NPs showed a time-dependent pattern and nearly reached a plateau three hours later. The green fluorescence of Co-6-labeled PTX-DPG NPs appeared in the cytoplasm of the Hela cells and maintained a high level for a long time. Microtubules and microfilaments are protein-fiber reticulation systems distributed in the cytoplasmic matrix, and there is a dynamic equilibrium between microtubules and microtubulin dimers under normal conditions. PTX could destroy this dynamic balance to exert anti-tumor effects; it could prevent the tumor cells from forming spindle bodies and spindle filaments when undergoing mitosis and then achieve the purpose of inhibiting cell division and proliferation. This result indicated that Co-6-labeled-PTX-DPG NPs were mainly distributed in the cytoplasm, where the PTX worked.

### 3.10. Biodistribution in Mice

To further illustrate the tumor-targeting effect of DPG and PTX-DPG NPs in vivo, three Hela tumor-bearing mice were dosed with DiR-labeled DPG micelles and PTX-DPG NPs and imaged.

**Figure 7:**
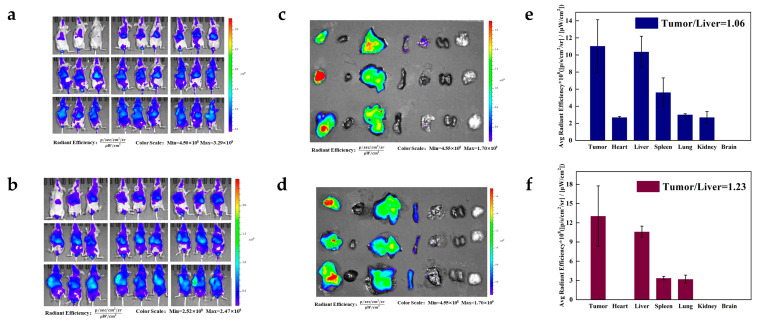
The in vivo biodistribution of DiR-labeled DPG micelles and PTX-DPG NPs in Hela tumor-bearing mice. Dynamic in vivo bio-distribution of Dir-labeled DPG micelles (**a**) and PTX-DPG NPs (**b**) at different time points (from left to right: 0.2 h, 0.5 h, 1 h, 2 h, 4 h, 6 h, 8 h, 12 h, and 24 h) post-dose through the tail vein. The fluorescent images of the tumor and major organs of mice receiving DiR-labelled DPG micelles (**c**) and PTX-DPG NPs (**d**) 24 h post-dose (from left to right: tumor, heart, liver, spleen, lung, kidney, and brain) (*n* = 3, mean ± SD) and their Fluorescence semi-quantitative analysis (**e**,**f**).

As seen in Figure 7a,b, it was clear that DPG micelles and PTX-DPG NPs mainly accumulated in the liver and tumor. Especially, PTX-DPG NPs show higher fluorescence in the tumor than in the liver at the 8th hour post dose. Since then, the fluorescence was continuously maintained at a high level in tumors, whereas it quickly declined in the liver. The fluorescence imaging of the dissected organs and tumors (Figure 7c,d) and the further quantitative analysis (Figure 7e,f) more clearly proved both DPG micelles and PTX-DPG NPs were mainly distributed in the liver and tumor, while PTX-DPG NPs had better tumor-targetability (the fluorescent intensity ratio of tumor/liver, 1.06 vs. 1.23). High tumor accumulation was supposed to be the main reason that PTX-DPG NPs demonstrated significantly improved anti-tumor efficacy in comparison with free PTX.

### 3.11. In Vivo Anti-Tumor Efficacy and Safety Evaluation

**Figure 8:**
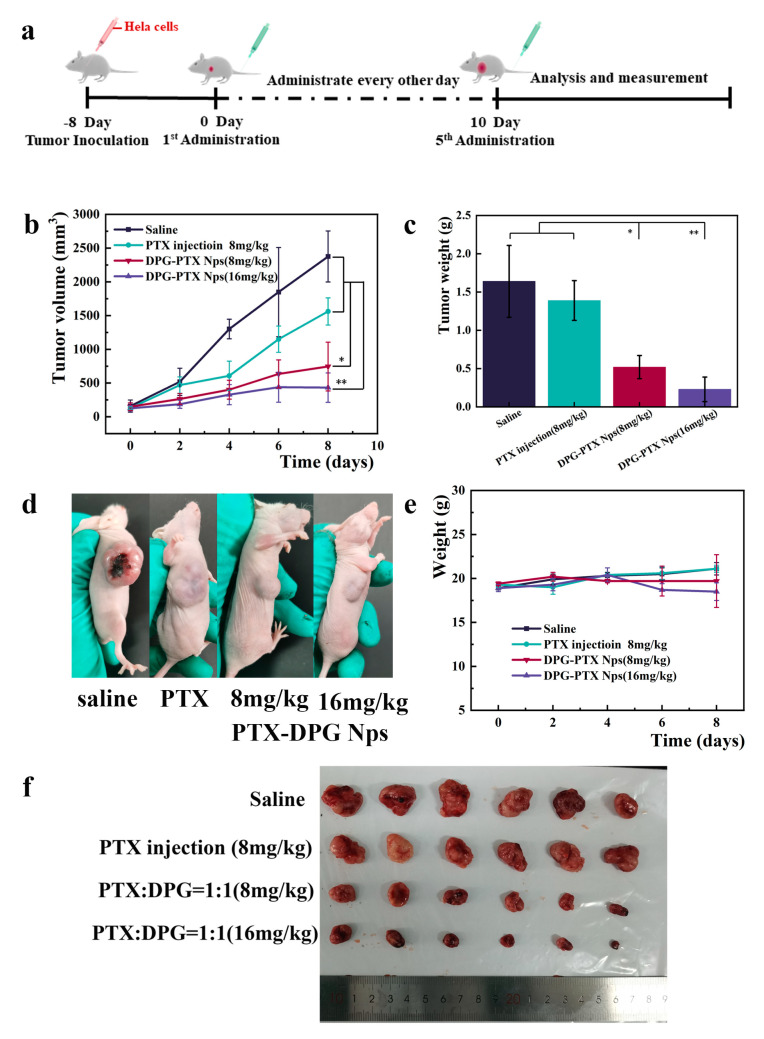
Schematic illustration of treatment of chemotherapy to Hela tumor−bearing mice (**a**). The mean tumor volume change (**b**) and the average tumor weight (**c**) of mice in each group. The results are presented as the mean ± SD, *n* = 6. ** *p* < 0.01 vs. PTX injection or normal saline, * *p* < 0.05 vs. PTX injection or normal saline. The photo of a representative mouse (**d**) and the average body weight change (**e**) for each group. (**f**) The photo of dissected tumors of all mice.

BALB/c nu/nu mice bearing Hela cells were selected to evaluate the therapeutic effect. The administration regimen was illustrated in Figure 8a. The profiles of tumor volume change in mice in groups were shown in Figure 8b, and the average tumor weights were shown in Figure 8c.

As seen in Figure 8a, the tumor of mice in the normal saline group grew rapidly, reaching 2375.94 mm^3^ at the end of the experiment. In contrast, the tumor of mice treated with PTX injections grew much slower, and PTX-DPG NPs treatment resulted in even slower tumor growth (*p* < 0.05), reaching 745.09 mm^3^ at the end of the experiment. It was no doubt that a high dose of PTX-DPG NPs treatment (16 mg/kg equivalent PTX) led to the smallest tumor (*p* < 0.01) (Figure 8b,d,f).

When the experiment came to an end, tumors were dissected, and accurately weighed (Figure 8c,f), based on which the tumor inhibition rate (TIR) was calculated (Table 2). Free PTX, namely PTX injections, only had a low TIR of 24.07 ± 2.84%, whereas the same dose of PTX-DPG NPs (8 mg/kg equivalent PTX) displayed a much higher TIR of 68.48 ± 9.99, demonstrating greatly improved therapeutic efficacy. The higher the dose (16 mg/kg), the higher the TIR (86.13 ± 9.6%).

It has been nearly 30 years since Taxol^®^, the first PTX dosage form, came into the market. PTX, due to its strong killing effect on a variety of malignant tumors and its relatively good tolerance, is still a powerful clinical anti-tumor drug with good development prospects. So far, many nanoparticle-based drug delivery systems, including polymer micelles, liposomes, microemulsions, nanoparticles, nanocrystals, cyclodextrin inclusions, and so on, have been explored to improve the safety and/or therapeutic efficacy, with the drug-loading content varying from 5% to about 90% (nanocrystals) [1]. High drug content reduced the need for chemical carriers, therefore decreasing the potential toxic side effects and immunogenicity induced by the excipients, especially after long-term administration.

In this study, PTX-DPG NPs achieved a drug-loading content of 41.45%, but that does not mean that 41.45% was a limit for PTX-DPG NPs. In the preliminary experiment, PTX-DPG NPs were successfully prepared at a PTX/DPG feeding ratio of 5:1, and the resultant mean particle size was about 234 nm, demonstrating a huge drug loading capacity for further encapsulation of other drugs. In contrast to PTX injections (24.07%), PTX-DPG NPs (8 mg/kg) reached a significantly improved tumor inhibition rate (TIR) of 68.48% (2.8-fold). The double dose (16 mg/kg) further elevated the TIR to 86.13% but failed to eradicate the tumor.

**Figure 9:**
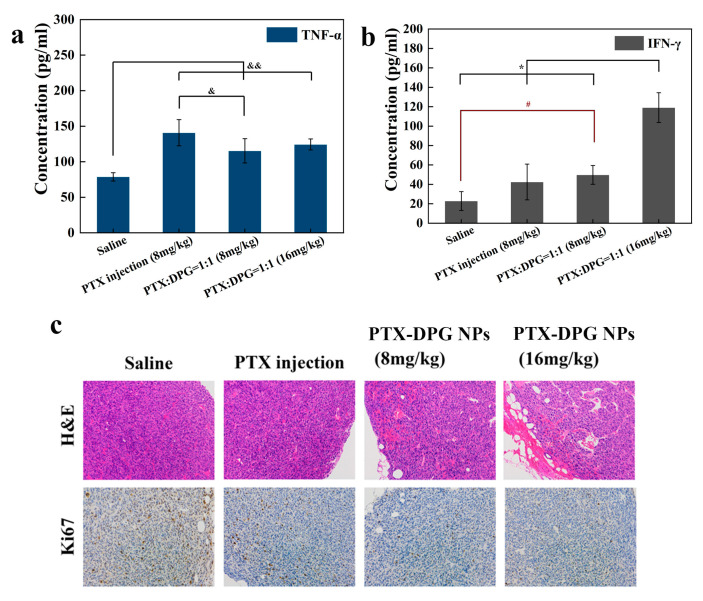
(**a**) Tumor cytokines of serum TNF-α level in the mice treated with saline, PTX injection (8 mg/kg), PTX-DPG NPs (8 mg/kg and 16 mg/kg of PTX) (Mean ± SD, n = 3). The results are presented as the mean ± SD, n = 6. * *p* < 0.01 vs. PTX-DPG NPs (16 mg/kg), ^#^ *p* < 0.05 vs. PTX-DPG NPs (8 mg/kg), ^&&^ *p* < 0.01 vs. saline, ^&^ *p* < 0.05 vs. PTX-DPG NPs (8 mg/kg). (**b**) Tumor cytokines of IFN-γ in the serum of mice treated with saline, PTX injection (8 mg/kg), PTX-DPG NPs (8 mg/kg and 16 mg/kg of PTX) (Mean ± s.d., n = 3). (**c**) H&E and Ki67 staining of tumor tissues in each group (Scale: 200×).

TNF-α was a cytokine that induced hemorrhagic necrosis in cells of tumor tissues. An increased serum TNF-α level meant a stronger killing ability on tumor cells. IFN-γ was another cytokine with anti-tumor and immunomodulatory effects. As shown in Figure 9a, all the mice administrated with free paclitaxel or PTX-DPG NPs showed a higher serum TNF-α level than those mice treated with normal saline, and free PTX was more effective than PTX-DPG NPs in this regard. In the case of TNF-α, PTX-DPG NPs seemed to be a stronger inducer than free PTX, especially at the high dose of 16 mg/kg (*p* < 0.01, in contrast to free PTX or 8 mg/kg of PTX-DPG NPs). The highly expressed TNF-α and IFN-γ NPs levels indicated the preferable property of inhibiting tumor cell proliferation.

Meanwhile, the apoptotic and proliferation pathways in tumors were also examined through pathological analysis and immunohistochemical studies. Hematoxylin and eosin (H&E) staining indicated that tumors treated with PTX-DPG NPs had much more porous tumor stroma, indicating tumor cell damage (Figure 9c). Ki-67 was the most commonly used proliferation marker. It was obvious that PTX-DPG NPs treatment led to a lower proportion of Ki-67-positive cells and a lower tumor cell proliferation rate than free PTX treatment.

Biosafety is very important for nanomedicine. Throughout the whole process of the anti-tumor efficacy study, repeated intravenous injections of PTX-DPG NPs at the dose of 8 mg/kg equivalent PTX were relatively well-tolerated with a little body weight loss and normal mice behavior, and the higher dose (16 mg/kg) of PTX-DPG NPs induced a little more body weight loss (Figure 8e). As illustrated in Table 2, there was no significant statistical difference in liver index among all groups, whereas PTX-DPG NPs induced a much lower spleen index (*p* < 0.05) than PTX injection, and the higher the dose, the lower the spleen index, indicating there was some splenic toxicity that needed to be overcome.

On the whole, PTX-DPG NPs greatly improved the therapeutic efficacy in Hela tumor treatment in contrast to PTX with acceptable tolerance; thus, it is promising to be a novel and effective anti-tumor agent for future application in the clinic.

## 4. Conclusions

In order to achieve optimal tumor therapy, nanomedicines are required to perform well in each of the five cascading steps after intravenous administration, including the long circulation in the blood, the effective accumulation in the tumor, penetration into tumor depth, internalization into tumor cells, and release of the encapsulated drugs [33]. Nanoparticles with surface charge reversal behavior are particularly advantageous in this respect because they can well balance the need for the negative charge of the nanoparticles in the blood circulation as well as the positive charge requirement of the nanoparticles as they traverse the tumor capillaries and permeate into the tumor depth. In this study, the charge reversal of PTX-loading nanoparticles was realized on the basis of DSPE-PEG-GSH (DPG), whose GSH moiety could be hydrolyzed by the GGT enzyme to expose the -NH2 group in the tumor site. The resultant PTX-DPG NPs showed small particle sizes, high drug-loading content, and good stability. PTX-DPG displayed good in vivo tumor accumulation and significantly improved therapeutic efficacy against breast tumors. DPG-based charge reversal of nanomedicine proved to be a good strategy for tumor-targeted drug delivery and was believed suitable for other anti-tumor agents in the treatment of many tumors with highly expressed GGT enzyme. However, the speed and degree of charge reversal of nanoparticles in this study still need to be further improved, and related research is already underway in our laboratory.

## Figures and Tables

**Table 1:** Surface element analysis of PTX-DPG NPs.

Elements	PTX Powder	PTX-DPG NPs
C	75.86%	38.88%
N	1.48%	10.57%
O	22.09%	49.37%
S	0%	1.17%

**Table 2:** In vivo anti-tumor efficacy and safety evaluation.

Group	Tumor Weight (g)	Tumor Inhibition Rate (%)	Liver Index ^@^	Spleen Index ^@^
Saline	1.64 ± 0.47		0.0624 ± 0.0037	0.0084 ± 0.0032
PTX injection (8 mg/kg) i.v.	1.39 ± 0.26	24.07 ± 2.84	0.0615 ± 0.0077	0.0082 ± 0.0015
PTX-DPG NPs (8 mg/kg) i.v.	0.52 ± 0.15 ^#&^	68.48 ± 9.99	0.0627 ± 0.0055	0.0051 ± 0.0005 ^$%^
PTX-DPG NPs (16 mg/kg) i.v.	0.23 ± 0.16 ^#&^	86.13 ± 9.6	0.0641 ± 0.0046	0.0039 ± 0.0002 ^#%^

The results are presented as the mean ± SD, n = 6. ^#^ *p* < 0.01 vs. normal saline. ^&^ *p* < 0.01 vs. PTX injection (8 mg/kg). ^$^ *p* < 0.05 vs. normal saline. ^%^ *p* < 0.05 vs. PTX injection (8 mg/kg). ^@^ Liver/spleen index is the ratio of the weight of the liver/spleen to the body weight of an experimental animal.

## Data Availability

Not applicable.

## Supplementary Materials

The following supporting information can be downloaded at: https://www.mdpi.com/article/10.3390/pharmaceutics15051335/s1, Figure S1: Transmission Electron Microscopy image of PTX-DPG NPs with the measured length and width.
