# Supplementary material for: A γ-Glutamyl Transpeptidase (GGT)-Triggered Charge Reversal Drug-Delivery System for Cervical Cancer Treatment: In Vitro and In Vivo Investigation
Source: Pharmaceutics. 2023 Apr 25;15(5):1335. doi: 10.3390/pharmaceutics15051335 (PMC10221838; doi:10.3390/pharmaceutics15051335)
Supplement: Supplementary file 1 [file pharmaceutics-15-01335-s001.zip › pharmaceutics-2238754-supplementary.pdf]

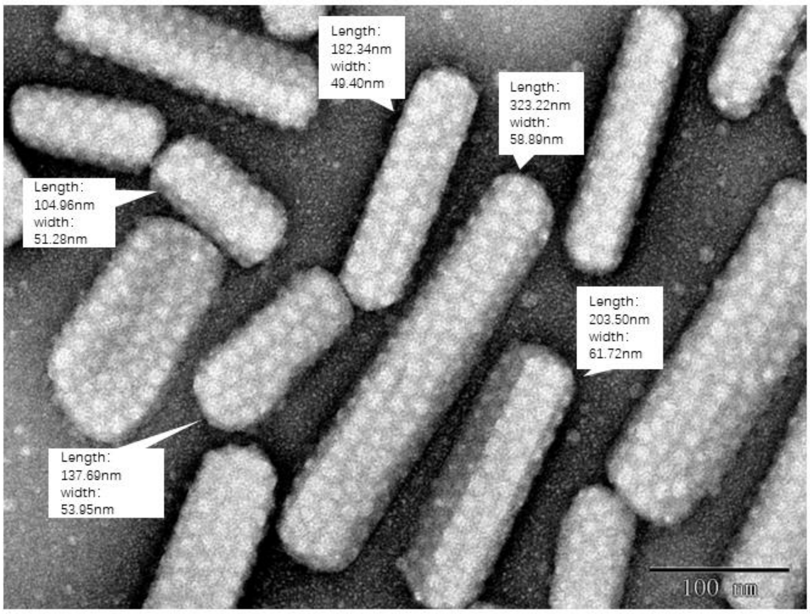

Figure S1: Transmission Electron Microscopy image of PTX-DPG NPs with the measured length and width.
